# Can cleaning processes based on ozone be used for high-touch surfaces in nursing homes in areas critical for infection control?

**DOI:** 10.3205/dgkh000518

**Published:** 2024-11-26

**Authors:** Anne Marcic, Axel Matthiessen, Albert Nienhaus, Jürgen Gebel, Carola Ilschner, Britt Hornei, Axel Kramer

**Affiliations:** 1 Public Health Department of the State Capital Kiel, Germany; 2 Institute of Hospital- and Environmental Hygiene, University Clinic Schleswig-Holstein, Kiel, Germany; 3 Institution for Statutory Accident Insurance and Prevention in the Health and Welfare Services (BGW), Hamburg, Germany; 4 Association for Applied Hygiene, c/o Institute for Hygiene and Public Health, University Hospital, Bonn, Germany; 5 Clinical Hygiene and Institute for Laboratory Medicine and Clinical Microbiology, Evangelical Hospital Oberhausen, Germany; 6 Institut of Hospital Hygiene and Environmental Medicine, University Medicine Greifswald, Germany

**Keywords:** ozone, decontaminating surface cleaning, biocide regulation, inhalation toxicity, occupational safety

## Abstract

In terms of infection control, environmental cleaning is critical in nursing homes, including long term care facilities. According to the statement of the Commission of Hospital Hygiene and Infection Prevention (KRINKO) at the Robert Koch Institute Berlin on the requirements for disinfectants in these areas, procedures should be used that have been certified by the Association for Applied Hygiene (VAH) for the necessary spectrum of efficacy (or are listed accordingly in the disinfectant list of the Robert Koch Institute).

Since ozone is a powerfully oxidizing gas with high inhalation toxicity, the conditions of ap-plication and the measures for occupational safety – including ensuring that the limit value in indoor air is not exceeded when handling and using the product –, must be declared by the manufacturer and observed by the staff to exclude toxic long-term hazard.

## Abstract (de)

Pflegeheime zählen zu infektionshygienisch sensiblen Bereichen. Gemäß der Stellungnahme der Kommission für Krankenhaushygiene und Infektionsprävention (KRINKO) am Robert Koch-Institut Berlin zu den Anforderungen an Desinfektionsmittel in diesen Bereichen sollen hier Verfahren zur Anwendung kommen, die vom Verbund für Angewandte Hygiene (VAH) für die jeweils notwendigen Wirkspektren zertifiziert wurden (oder ggf. entsprechend in der Desinfektionsmittel-Liste des Robert Koch-Institut aufgeführt sind).

Da Ozon ein stark oxidierendes Gas mit hoher Inhalationstoxizität ist, müssen bei Einsatz von Produkten auf Ozonbasis die Anwendungsbedingungen und die Maßnahmen zum Arbeitsschutz einschließlich der Gewährleistung einer fehlenden Überschreitung des Grenzwerts in der Raumluft beim Umgang mit dem Produkt und bei seiner Anwendung herstellerseitig deklariert sein und durch das Personal beachtet werden, um eine toxische Langzeitgefährdung auszuschließen.

## Infection risk in nursing homes and consequences for disinfecting surface cleaning

Aging is associated with age-related organ changes, immunosenescence, inflammaging, comorbidities, geriatric syndromes, frailty, and malnutrition. Associated with the transmission possibilities of pathogens in nursing homes, infections of residents are a significant cause of morbidity and mortality [1], [2], [3], [4], [5], [6], [7]. Disinfecting surface cleaning is part of an infection prevention bundle in nursing homes [8]. For hard surfaces requiring disinfection according cto the risk-assessment approach described by the Commission of Hospital Hygiene and Infection Prevention (KRINKO) [9], disinfectants should be used whose efficacy has been demonstrated by two manufacturer-independent test reports and corresponding expert opinions from testing laboratories which also confirm the specific application recommendation.

Regarding efficacy and spectrum of activity, an assessment of the infection risk is necessary for the selection of suitable cleaning and disinfection procedures. For this, the KRINKO recommendation for cleaning and disinfection of surfaces provides detailed guidance [9]. For high-touch surfaces in nursing homes, disinfecting cleaning or, for surfaces used for aseptic activities, disinfection is indicated depending on the situation. Disinfecting cleaning primarily refers to cleaning and disinfection in one step [10]. According to the KRINKO statement on the requirements for disinfectants in these areas [10], procedures that have been certified by the VAH for the necessary spectrum of activity should be used here. The required spectrum of activity, which must be bactericidal and levurocidal (effective against yeasts) at a minimum, and possibly also tuberculocidal, mycobactericidal, fungicidal, sporicidal and/or virucidal, results from the risk assessment.

## Disinfectant cleaners based on ozone

In Germany, unlike disinfectants, one-step disinfectant cleaners are not a legally defined category of their own. According to their intended use and mode of action as products for disinfection, they must hence be approved as a biocide or registered as medical device [11].

Ozone generated from oxygen, which includes the generation of ozone from atmospheric oxygen, technical oxygen or water, has been approved as a biocidally active substance in the Biocidal Products Regulation, R4BP asset number EU-0029722-0000 from July 1, 2024, for the application of product group PT02 (disinfectants and algaecides not intended for direct application to humans and animals) [12]. However, the assessment documents for product group PT02 do not explicitly list methods for the disinfection of surfaces (hard surfaces) using stabilized ozone-containing water. Currently there is no approved biocidal product based on ozone as an active substance.

Several products for the decontamination of surfaces are offered on the market with content of ozone, generated in circulating water by special electrodes. These products, based on stabilized ozone-containing water, are neither an approved biocidal product nor certified and listed as disinfection procedures by VAH [13]. They are not marketed as biocides or certified disinfectants, but instead described as sanitizing systems “with germ-reducing effect”. However, sanitizers do not meet the requirements for a disinfectant or a one-step disinfectant cleaner. Such a product can only be used for the depletion of microorganisms, viruses, and contaminants by damp wiping.

Even if a procedure shows effectiveness against individual pathogens or test organisms that corresponds to disinfection (e.g., a reduction in the number of a bacterial species by 5 lg, which corresponds to a reduction of 99.999%, or reduction of a virus by 4 lg, corresponding to a reduction of 99.99%), this does not mean that the test criteria for a disinfection procedure according to VAH or European standards are met. Use in the healthcare sector, including nursing homes, is only permissible if the disinfectant effectiveness for the required spectrum of activity (e.g., bactericidal, yeasticidal) has been confirmed.

Currently, automated room disinfection using ozone fumigation with mobile devices is not recommended by KRINKO in health facilities [10], because the evaluation of various ozone-based technical methods has not yet been completed. A recent review by a group of authors from Great Britain and the USA [14], [15] came to the same conclusion.

## Avoiding negative side effects of ozone-based disinfecting systems

In addition to the effectiveness of ozone-based agents or procedures, their tolerability must be considered depending on the mode of application, since ozone is a strongly oxidizing gas with high inhalation toxicity.The manufacturers must indicate whether ozone can be released during the use of an ozone-based sanitizer on surfaces or whether the ozone decom-poses during application without resulting in inhalative exposure to ozone. To ensure the stability of ozone products, the use of stabilizing additives is necessary. The volatility of ozone must be specified for each product.

Due to the high inhalation toxicity, the threshold values in the ambient air must not be ex-ceeded. Since no occupational exposure limit (OEL) for ozone has yet been established, the existing MAK value (Maximum Workplace Concentration) or international threshold values of 0.12 mg/m³ [16] serve as a guideline for workplace concentration. The DNEL (Derived No-Effect Level), which describes the exposure limit below which a substance is not expected to impair human health according to scientific knowledge, can also serve as guidance. The DNEL for ozone is 0.024 mg/m³ (GESTIS-DNEL list [17]). Values issued by the Swiss State Secretariat for Economic Affairs (SECO), Department of Basic Labor and Health Issues (ABGG), also provide guidance [17]. According to the latter, the average concentration of ozone over the duration of a day’s work (8 h) should not exceed 35 ppb. As a short-term limit (15 min), a maximum of 4x15 minutes up to 60 ppb is allowed at intervals of 1 hour daily.

It must also be ensured that there is no penetration of ozone through the protective gloves worn when performing surface disinfection. This pertains to skin contact and not to protection against any gaseous ozone that may be released by the solution. Since stabilized aqueous ozone solutions act microbicidally, ozone is present as a highly reactive agent. Skin damage is not to be expected at the concentrations used in these sanitizers. However, if ozone penetrates the glove and is absorbed in traces, a risk from long-term exposure over a professional lifetime cannot be ruled out, as gaseous ozone can induce oviductal carcinomas in B6C3F1 mice [18]; consequently, it is justified to suspect a carcinogenic potential in case of inhalative exposure. Whether this also applies to skin contact is unknown. However, for safety reasons, it is recommended to wear special protective gloves with additional so-called antiozonants when handling stabilized aqueous ozone solutions. For example, protective gloves made of butyl rubber exist that have ozone-resistant properties [19].

## Necessary preconditions for using ozone-based methods

Users should have information on the following questions:

- Is the ozone used in gaseous form or in aqueous solution? (For example, “SAO” means stabilized aqueous ozone, i.e., water-based, see also current studies on the various methods ([14], [13])?
- How is the stabilization of volatile ozone in the product achieved?
- What is the intended use specified by the manufacturer?
- For which purposes is the method possibly approved as a biocide or medical device for disinfection?
- Has the effectiveness against the indicated spectrum of activity (e.g., bactericidal, levurocidal, fungicidal, tuberculocidal, mycobactericidal, sporicidal or virucidal) been confirmed by two independent and accredited test laboratories with expert opinions?
- Which surfaces can be treated with the method in the intended facility, based on risk assessment?
- What are the requirements for application in terms of preparation, training for implementation, post-processing, and occupational safety for the respective method?

## Conclusions

The approval date for the biocidally active substance ozone generated from oxygen is 01.07.2024. Applications for approval must be submitted by this date to one of the competent authorities, e.g., the Federal Authority for Chemicals in Germany in accordance with Registration, Evaluation, Authorisation and Restriction of Chemicals (REACH), and will then be reviewed by the authorities.

If the indication for disinfecting surface cleaning or surface disinfection according to the KRINKO recommendation [9] exists, the application of a method is only justifiable if the product is approved as a biocide or registered as a medical device and the efficacy has been confirmed and certified according to the requirements of the VAH or the EN requirements in two manufacturer-independent expert opinions. This has not yet been achieved for methods based on stabilized ozone-containing water.

When using ozone-based products, the conditions of application and the measures for occu-pational safety during handling of the product and its application must be declared by the manufacturer and observed to exclude toxic long-term hazards by this highly reactive sub-stance. Additionally, possible risks should be considered by the employer as part of risk assessment in accordance with the Hazardous Substances Ordinance. and protective measures should be taken to exclude any risk to the health of employees.

## Notes

### Competing interests

The authors declare that they have no competing interests.

### Authors’ ORCID 

• Anne Marcic: 0009-0008-3660-040X

• Axel Kramer: 0000-0003-4193-2149

• Albert Nienhaus: 0000-0003-1881-7302

• Jürgen Gebel: 0000-0001-9328-3174
